# Demographic, socioeconomic and functional health-related factors in the selection of vaginal pessaries used for the conservative treatment of pelvic organ prolapse: a retrospective study

**DOI:** 10.1186/s12905-025-03923-9

**Published:** 2025-08-13

**Authors:** Orsolya Oláh, Miklós Romics, Nándor Ács, Richárd Cseh, Péter Nyirády, Attila Majoros

**Affiliations:** 1 Department of Obstetrics and Gynecology, Semmelweis University, Üllői út 78/A, Budapest, H-1082 Hungary; 2 Department of Urology, Semmelweis University, Üllői út 78/B, Budapest, H-1082 Hungary

**Keywords:** Pelvic organ prolapse, Conservative therapy, Vaginal pessary, Cube pessary, Ring pessary

## Abstract

**Background:**

Vaginal pessary therapy is a well-known conservative treatment of pelvic organ prolapse (POP). According to several national guidelines, pessary therapy should be used as a first-line treatment for pelvic organ prolapse. A distinction is made between pessaries that can be worn permanently or require daily control. This therapy is primarily chosen by elderly patients who are not suitable for surgery or by patients not wishing to undergo surgical treatment. The aim of our study was to investigate what demographic, socioeconomic and functional health-related factors play a role in the choice of the pessary types, and to provide healthcare professionals with guidance on recommending a pessary type to patients based on these factors.

**Methods:**

115 female patients using vaginal pessaries for POP were included in our study (group I:73 pts used a daily control “cube” pessary (DCP), group II.:42 pts used a long-term wear ring pessary (LTP). Data collection was performed between March 2021 and March 2022 through a personally completed questionnaire or telephone interview. The following factors were investigated: patients’ age, fertility, education level, employment status, marital status, independence, hand function, vision, mobility, physical and sexual activity.

**Results:**

A significant difference was found between the DCP and LTP groups in terms of average age (44.9 vs. 75.7 years *p* < 0.0001), fertility (61.6 vs. 4.6% *p* < 0.0001), employment status (retired: 18 vs. 100% *p* < 0.0001), marital status (single: 8.3 vs. 44.1% *p* < 0.0001) and educational level (highest: 81.9 vs. 25.6% *p* < 0.0001). In terms of functional health-related factors, such as weakened hand function (1.4 vs. 34.9% *p* < 0.0001), vision impairment (19.4 vs. 58.1% *p* < 0.0001), reduced mobility (5.5 vs. 81.4% *p* < 0.0001), sexual activity ratio (87.5 vs. 0% *p* < 0.0001), and the level of physical activity (VAS: 9.5 vs. 6.1 *p* < 0.01) also significant differences were registered between the DCP and LTP groups.

**Conclusions:**

The choice of the type of vaginal pessary treatment for POP is influenced by many patient-side factors.

**Trial registration:**

The study was conducted with the approval of the Regional Institutional Scientific and Research Ethics Committee of Semmelweis University (SE RKEB 97/2022).

## Background

Pelvic Organ Prolapse (POP) refers to the descent of the uterus, vaginal walls, and consequently the urethra, bladder, and rectum or small bowels due to weakening and stretching of the endopelvic fascia system that forms part of the pelvic floor [1]. There is a wide range of surgical techniques available for the treatment of pelvic organ prolapse (POP), encompassing variations in approach, materials, and specific procedures. Surgical options for pelvic organ prolapse (POP) are associated with limitations such as recurrence rates and complications, making conservative treatments, including pessary use, an essential alternative for many patients. Conservative management is particularly important given the restrictions and risks associated with vaginal implants, as well as the recurrence rates observed after various surgical procedures. With the restrictions on vaginal implants, techniques that were long used, such as sacrospinous ligament fixation (Amreich-Richter operation), which allows vaginal apical suspension without a vaginal implant, have become popular again [2]. Currently, sacrocolpopexy (whether it be laparoscopic, robot-assisted, or even abdominal) is the most effective method for isolated apical suspension. However, it’s important to note that the recurrence rate for sacrocolpopexy is also noteworthy, depending on the study, around 10–22% [3, 4], with the outstanding success rate visible in cases of isolated apical prolapse [5]. During the COVID-19 pandemic, the preference for vaginal surgeries increased due to their feasibility under regional anesthesia and avoidance of aerosol-generating procedures, which highlights the importance of adaptable surgical approaches. However, conservative treatments like pessary therapy remain critical alternatives for managing POP, especially in patients unsuitable for surgery [6].

Many people reject surgical solutions due to old age, poor general condition, and even if they undergo an operation, many of them have a strong fear of surgery [7]. Moreover, In the French guideline on pelvic organ prolapse you can find that for symptomatic pelvic organ prolapse, pessary should be offered to every patient as a first-line treatment regardless of the patient’s age and the stage of the prolapse [8]. The Canadian guideline on pessaries used for pelvic organ prolapse also emphasises, that in most cases, it is possible to find a pessary that suits the patient, although certain anatomical or functional factors may limit the success of pessary fitting. Based on these findings, they recommend that every woman suffering from symptomatic pelvic organ prolapse try a pessary [9]. The above guidelines are also supported by a prospective study in which the efficacy of pessaries and reconstructive surgeries were compared among patients with symptomatic prolapse. At a one-year follow-up, according to the Sheffield Questionnaire comparison of the two groups demonstrated no significant differences in prolapse, bladder, bowel and sexual function symptoms, apart from frequency of intercourse which was better in the surgery group (54% vs. 46%; *p* = 0.028) [10].

Pessaries– already used in ancient Egypt for the treatment of prolapse [11] - can be classified into two main groups. Long-term wear pessaries (LTP) remain in the vagina permanently after insertion and are only removed during specialist visits and their most common form is the ring pessary. The other type is the daily control pessary (DCP), which patients insert in the morning and remove before going to bed. Daily control pessaries (DCP) have the advantage that, unlike long-term wear pessaries (LTP), they do not create pressure points in the vagina where vaginal wall erosions could occur due to prolonged reduced blood supply. The most common form is the cube pessary [12]. While pessary therapy is well-established as a first-line treatment for POP, limited research exists on how individual patient characteristics determine the selection between long-term wear pessaries and daily control pessaries. Our hypothesis was that a combination of demographic, socioeconomic, and functional health-related factors influence the choice of pessary type, with younger, sexually active patients preferring daily control pessaries and older, less mobile patients opting for long-term wear pessaries.

The aim of our study was to confirm this hypothesis and determine the factors that influence patients’ choices and to address a critical gap in understanding the demographic, socioeconomic, and functional health-related factors influencing the choice of pessary type for the conservative management of pelvic organ prolapse (POP). While pessary therapy is well-established as a first-line treatment for POP, limited research exists on how individual patient characteristics determine the selection between long-term wear pessaries and daily control pessaries. This study provides novel insights by evaluating factors such as hand function, vision, mobility, and sexual activity, which have not been systematically explored in previous literature.

## Methods

A total of 115 female patients, including 73 using daily control pessaries (DCP) and 42 using long-term wear pessaries (LTP) continuously were selected in our retrospective study. Inclusion criteria were: (1) female patients diagnosed with pelvic organ prolapse (POP), (2) undergoing treatment with a vaginal pessary (either daily control or long-term wear), (3) able to provide informed consent, and (4) willing and able to complete the questionnaire or telephone interview. Exclusion criteria were: (1) incomplete data in the questionnaire or telephone interview, (2) patients not actively using a vaginal pessary at the time of the study, and (3) significant cognitive impairments or language barriers that prevented effective participation in the study. Patients were retrospectively identified using convenience sampling from a clinical database of individuals who had used vaginal pessaries for pelvic organ prolapse (POP). Data were collected between March 2021 and March 2022 using a standardized questionnaire with single-choice answers. Patients who returned for control exams completed the questionnaire on paper, while those who did not attend in person were contacted by telephone and asked the same questions in a standardized format to ensure consistency in responses. While convenience sampling was employed, we recognize that this approach may introduce selection bias, which is a limitation of this study. Patients were retrospectively identified by reviewing their medical records, from which information such as POP-Q stages and duration of complaints was extracted. Additional data were newly collected through a standardized questionnaire administered during control exams or via telephone interviews. *p* = 0.34) At prolapse staging we used the Pelvic Organ Prolapse (POP–Q) Quantification system [13].

The following patients’ characteristics were investigated as potential factors affecting patients’ choice of pessary type: patients’ age, education level, employment, fertility and marital status, independence, hand function, vision, mobility, physical activity (VAS 0–10), and sexual activity. The variables included in the study, such as independence, physical activity, sexual activity, age, socioeconomic factors, and functional health-related factors (e.g., hand function and vision), were selected based on their potential to influence pessary choice. These factors were hypothesized to affect the feasibility of handling different pessary designs and reflect lifestyle considerations, such as the ability to manage daily insertion and removal or the desire to accommodate sexual activity. Independence was assessed using a binary question in the questionnaire, where patients were asked to choose between two options: (1) self-sufficient in daily life or (2) needs assistance. Physical activity was quantified using a scale of 0 to 10, where 0 indicated ‘completely inactive’ and 10 indicated ‘completely active.’ Patients rated their typical daily activity level based on this scale. We also inquired about the reasons for the patient’s choice of pessary therapy. Possible answers included: a preference to avoid surgery, prior unsuccessful POP surgery, or a decision based on a compromised general condition. The primary outcome of the study was to identify demographic (e.g., age, marital status), socioeconomic (e.g., education, employment status), and general health-related factors (e.g., fertility) influencing the choice between DCP and LTP among patients with pelvic organ prolapse (POP). Secondary outcomes included: (1) the impact of sexual activity on the choice of pessary type, (2) the role of physical skills (e.g., hand function, mobility, and vision impairments) in influencing the feasibility and preference for DCP versus LTP, and (3) patient-reported reasons for choosing pessary therapy, including a preference to avoid surgery, prior unsuccessful POP surgery, or a decision based on a compromised general condition.

The study was conducted with the approval of the Regional Institutional Scientific and Research Ethics Committee of Semmelweis University (SE RKEB 97/2022). Informed consent was obtained from all participants prior to their inclusion in the study. These procedures were conducted in compliance with ethical guidelines and approved by the ethics committee. For statistical analysis, all numerical variable data sets were subjected to the D’Agostino-Pearson normality test as a first step. In all cases normal distribution of the data was verified therefore, the *p* values were determined by Student’s unpaired *t* test. For the categorical variable data sets, Pearson’s chi-square (χ²) test was used. The statistical tests used were appropriate for the data, and the observed statistically significant results (*p* < 0.05) suggest sufficient power for detecting meaningful associations in this population.

## Results

The mean age of the patients was 51.4 ± 18.4 years. The average duration of pessary use was 26.7 ± 15.5 months (DCP group: 24.7 ± 12.5 vs. LTP group: 28.3 ± 23.6 months, The highest POP-Q stages in the groups were distributed as follows: LTP group: stage 1: 11,9% (5 patients), stage 2: 52,4% (22 patients), stage 3: 23,8% (10 patients), stage 4: 11,9% (5 patients). DCP group: stage 1: 6,8% (5 patients), stage 2: 63,0% (46 patients), stage 3: 24,6% (18 patients), stage 4: 5,5% (4 patients).

There was no significant difference (*p* < 0.31) in the duration of complaints between the DCP and LTP groups (3.4 ± 4.9 vs. 2.7 ± 2.9 years). The mean patients’ age was significantly lower in the DCP group than in the LTP group (44.9 ± 13.5 vs. 75.7 ± 6.9 years, *p* < 0.0001).

There was a significant difference in the reasons for choosing conservative treatment between the groups (Table 1.). In the DCP group, 97.2% (70 patients) chose the pessary to avoid or delay surgery, while in the LTP group, this proportion was only 27.9% (12 patients, *p* < 0.001). Previous unsuccessful surgery was reported in the history of 2.8% of the DCP group and 20.9% of the LTP group (*p* < 0.01). Poor general condition, which is a contraindication for surgery, was not present in the DCP group, whereas it was provided in 51.2% (22 patients) of the LTP group (*p* < 0.001).

**Table 1:** Comparison of reasons for choosing vaginal Pessary therapy. DCP: daily control Pessary, LTP: long term wear Pessary. Limit of significance: *p* < 0.05

Why did patient choose it?	Total *n* = 115 (100%)	DCP group *n* = 73 (100%)	LTP group *n* = 42 (100%)	*p*
Did not want surgery	82 (71.3)	70 (97.2)	12 (27.9)	*p* < 0.001
Previous unsuccessful surgery	11 (9.6)	2 (2.8)	9 (20.9)	*p* < 0.01
Poor general condition	22 (19.1)	0	22 (51.2)	*p* < 0.001

**Fig. 1:**
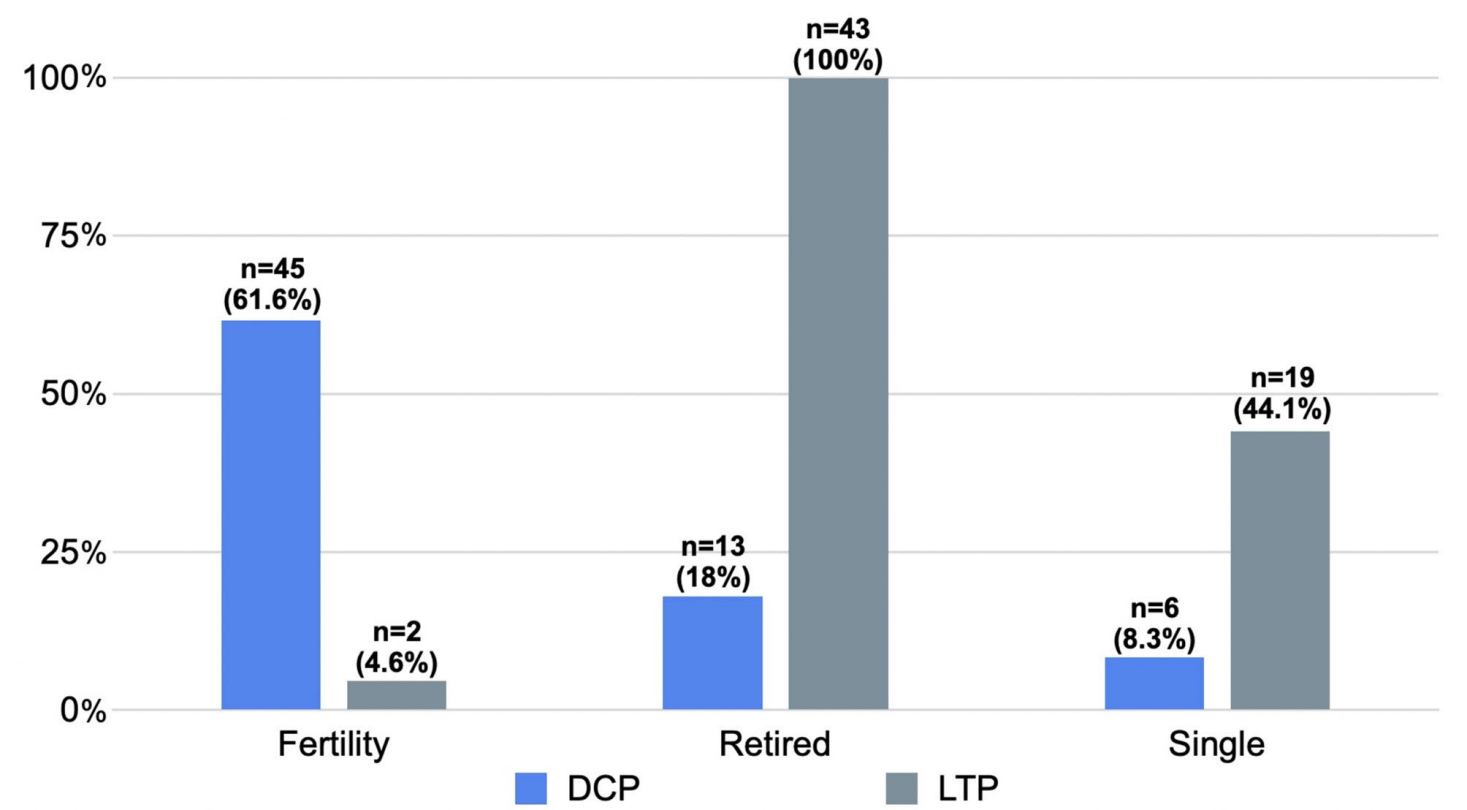
Comparison of fertility, marital and employment status between DCP (daily control pessary) and LTP (long term wear pessary) groups

Significant differences were also observed in terms of fertility, marital and employment status (Fig. 1). In the DCP group 61.6% of patients were of reproductive age, while only 4.6% was in the same status in the LTP group (*p* < 0.0001). In terms of marital status, only 8.3% of DCP users were single, while in the LTP group this percentage was 44.1% (*p* < 0.0001). In the DCP group only 8% of patients were retired, opposite to the LTP users this ratio was 100%(*p* < 0.0001).

The DCP group had a significantly higher proportion of people with tertiary education (81.9% vs. 25.6%, *p* < 0.0001). Secondary education was 13.9% in the DCP group and 51.6% in the LTP group, which is also a statistically significant difference (*p* < 0.0001). Primary education was significantly higher in the LTP group (23.2 vs. 4.2%, *p* = 0.017). The results for educational attainment are shown in Table 2.

**Table 2:** Comparison of educational levels between DCP (daily control pessary) and LTP (long term wear pessary) users significance level: *p* < 0.05

School education	Total *n* = 115 (100%)	DCP *n* = 73 (100%)	LTP *n* = 42 (100%)	*p*
Primary education	13 (8.7)	3 (4.2)	10 (23.2)	*p* = 0.017
Secondary education	32 (27.8)	10 (13.9)	22 (51.6)	*p* < 0.0001
Tertiary education	70 (60.8)	59 (81.9)	11 (25.6)	*p* < 0.0001

**Fig. 2:**
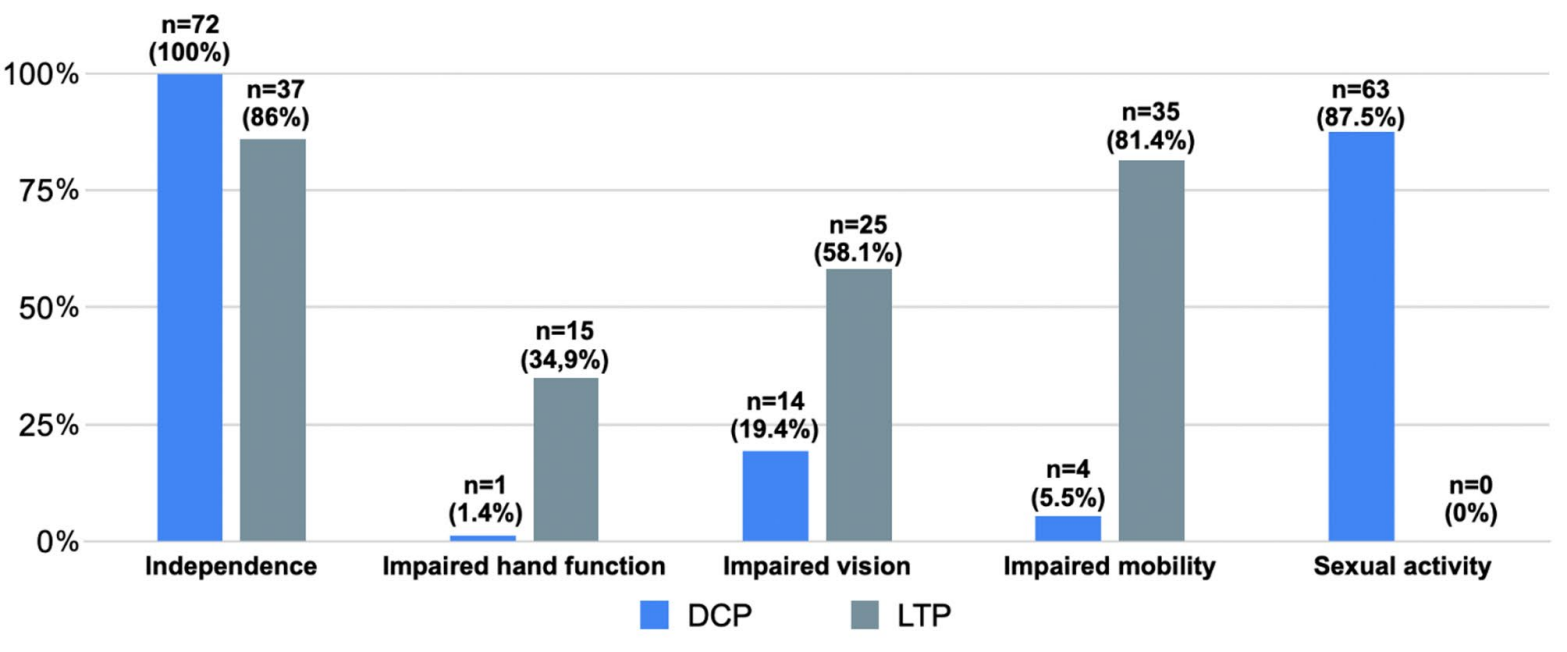
Comparison of lifestyle factors between DCP (daily control pessary) and LTP (long term wear pessary) users (significance level: *p* < 0.05)

The physical skills that influence pessary use are presented in Fig. 2. Although there was no significant difference in independence in activities of daily living between the DCP (100%) and LTP groups (86%), we observed significant differences in both hand and visual function and mobility, physical and sexual activity. Impaired hand function was seen in 34.9% of the LTP group and only 1.4% of the DCP group (*p* < 0.0001). Impaired vision was detected in 58.1% of the LTP group and 19.4% of the DCP group (*p* < 0.0001). Impaired mobility was observed in 81.4% of the LTP group and only in 5.5% of the DCP group (*p* < 0.0001). There was also a significant difference in daily activity on the 10-point scale of the visual analogue scale, with a mean activity of 9.5 ± 9.6 in the DCP group and 6.1 ± 1.9 in the LTP group (*p* < 0.01).

Sexual activity also differed significantly, with 87.5% of patients in the DCP group being sexually active compared to 0% of patients in the LTP group (*p* < 0.0001).

## Discussion

POP is a common condition which can cause different functional and nonfunctional symptoms and decrease the life quality of the patients [14]. Before surgical treatment the use of vaginal pessaries can be suggested as a first-line therapy [8, 9] to solve or improve the symptoms [15]. This study contributes to the field by systematically analyzing the interaction of demographic, socioeconomic, and functional health-related factors in influencing pessary choice. Unlike previous studies, which primarily focus on anatomical or clinical outcomes, our findings highlight the importance of factors such as sexual activity, functional impairments, and patient preferences, offering new perspectives for personalized pessary selection in clinical practice. This can be supplemented with pelvic floor exercises, but in more advanced prolapse stages (POP-Q stage II, III) pelvic floor exercises alone are not as effective as pelvic floor physiotherapy and vaginal pessary together [16]. The permanent pessariesare also known as supportive pessaries. Their most common complications are heavy vaginal discharge, vaginal bleeding, and vaginal wall erosion [17]. To prevent these issues, in our practice, proper pessary fitting (not too big size), regular gynecological check-ups every 3–6 months are recommended to monitor for early signs of erosion. If erosion occurs, management involves temporarily removing the pessary until the erosion resolves, typically supported by the use of vaginal estrogen to promote mucosal healing. Follow-up visits every 4 weeks during the healing process are advised, with reinsertion of the pessary considered once the erosion has completely healed, depending on the patient’s preference.

The other pessaries require daily control, and they have the advantage that, unlike long term wear pessaries, the above complications do not occur, vaginal intercourse is possible [12].

Despite their ancient history and diversity, some experts lack sufficient experience with pessaries and are uncertain about the choice of shape and size. This is supported by a recent French study in which only 54% of healthcare professionals would recommend pessaries as a first-line therapy for pelvic organ prolapse (POP) based on the responses of more than 1000 healthcare professionals [18]. The popularity of pessaries also depends on the location, for instance in a recent survey sent to urology and gynecology hospital departments in Hungary in 2022, 96.15% of specialists offered conservative therapy for POP, although the response rate of the departments included in the survey was only 24.76%, which may skew the results [19]. Among urogynecologists, the percentage of pessary knowledge is obviously higher, with 77% of members of the American Urogynecological Society (AUGS) recommending pessaries as first-line treatment [20].

Even if the intention to use a pessary is there, finding the right type and size can be difficult, as confirmed by a study which shows that the chance of pessary expulsion is 30–60%, which can be due to short vaginal length, wide introitus, or poor sizing among other reasons [21]. However, when the appropriate pessary is successfully found for the patient, quality of life can significantly improve, as demonstrated by a 2018 Chinese study in which 79% of 162 patients successfully fitted with a pessary reported satisfaction or high satisfaction [22]. A similar result was found in a Hungarian study, where an 84% improvement in quality of life was observed during follow-up of pessary users [23].

The patient’s sexual activity predominantly influences this decision, leading us to predominantly opt for daily control pessaries (DCP) for younger patients. However, to our knowledge, our study represents the first investigation on this specific topic, as demographic, socioeconomic and functional health-related factors in the selection of long-term wear and daily-control pessaries have not been investigated.

The available literature on the causes of failed pessary use has generally focused on a group of patients who have used a LTP, mainly ring pessaries. In one such study, sexually active women had a higher rate of unsuccessful LTP use (DCP was not studied) [24], consistent with our findings that sexually active women are more likely to choose daily control cube pessaries. In another study, older age was found to be a predisposing factor for unsuccessful pessary use [25]. A Chinese study found that for younger patients with a large levator hiatus, the Gellhorn pessary was the optimal choice over the ring pessary, but the daily control cube pessary was not investigated [26]. 97.2% of the DCP group chose to use a pessary to avoid surgery, compared to 27.9% in the LTP group. The former group was significantly younger and mostly of reproductive age with prolapse occurring after childbirth. In our study in the DCP group 91.2% were in a relationship, and 87.5% were sexually active. Sexual activity is a significant factor influencing pessary choice, particularly for patients with pelvic organ prolapse (POP), which is known to severely impact quality of life and sexual function. Daily control pessaries (DCP) allow for removal during intercourse, offering a distinct advantage for sexually active patients. While pessaries can help manage prolapse symptoms, further research is needed to assess their direct impact on sexual satisfaction and function.

Due to older age, only a very small proportion of LTP users (4.6%) was of reproductive age and nobody of these patients was active sexually. At the same time, it is important to emphasize that patients who choose a long-term wear pessary can still have a sexual life. It is generally possible to have sex with a ring pessary in place. However, comfort levels can vary from person to person. Due to occasional vaginal wall erosions associated with the use of a ring pessary, there might be a risk of vaginal wall injury during vaginal penetration with a ring pessary in place. Therefore, we primarily recommend a daily control cube pessary for those of our patients who wish to have a vaginal sexual life. In addition, in certain cases, we can also teach patients who use a ring pessary and wish to engage in vaginal sexual activity, with proper cooperation, how to remove the ring pessary before sexual intercourse and replace it afterwards. Even if penetration might not work with filling pessaries (for example donut), it’s not advisable to limit sexuality to vaginal penetration, meaning that intimacy and sexual encounters can still be experienced with a long-term wear pessary. In this group 20.9% of patients had already undergone one or more failed reconstructive surgeries and all of them were already retired, therefore exposed to lower levels of physical strain in their daily activities and less of them wanted to delay POP surgery compared to the DCP users (27.9% vs. 97.2%). The high POP surgery rate measured in the LTP group corresponds to the literature data and draws attention to the unresolved nature of POP treatment. In a Cochrane Systematic review, recurrent POP after anterior colporrhaphy occurred between 32 and 45% [27]. In another Cochrane Systematic review recurrent POP after posterior compartment surgeries occurred in 10% after transvaginal repair, and between 16% and 100% after transanal repair [28]. After apical prolapse surgeries recurrence according to a third Cochrane Systematic review occurred in 23% after sacral colpopexy and in about 41% (31–63%) after vaginal procedures [29].

The LTP group had significantly higher rates of impaired hand function, vision, and mobility, which may suggest that the lack or reduction of these functions discourages daily use of removable pessaries. It should also be noted that this older patient population had lower levels of physical and sexual activity compared to the DCP group, which may further contribute to the decision to use LTP.

The finding that DCP users are more likely to have tertiary education highlights the potential influence of education on pessary choice. Tertiary education is often linked to increased body awareness, greater confidence in managing health-related tasks, and improved access to healthcare resources. These factors may enhance the ability to independently manage daily removal and reinsertion of DCPs, aligning with the autonomy required for their use. This underscores the importance of considering educational background when tailoring pessary recommendations to individual patients.

The real primary practical purpose of the study was to provide guidance to the healthcare professionals on recommending a pessary type to patients based on their age, sexual activity, and other parameters we examined. These parameters do play a role in the patient’s choice of pessary type, but ultimately the decision lies with the patient.

One of the most significant findings of this study is the demographic and functional differences observed between DCP and LTP users. Younger, sexually active patients with greater physical independence and better hand function preferred DCPs, likely due to the flexibility and autonomy they offer. In contrast, older patients with impaired mobility or hand function were more likely to use LTPs, aligning with their reduced ability to manage daily removal and reinsertion. These insights provide practical guidance for healthcare professionals, emphasizing the importance of considering both demographic and functional characteristics during pessary selection.

One of the strengths of our study is that it focuses on daily-use pessaries, including the cube pessary, which has received limited attention in the literature with only 9 relevant publications found on PubMed in the past 10 years. Based on the available literature, most institutions mainly use only long-term wear pessaries, and there is no option for individual pessary selection.

Another strength is that, to our knowledge, no previous studies have examined the factors we investigated (hand function, vision, mobility, or education level) in relation to the use of pessaries.

The weakness of our study is the lower number of cases, the heterogenity of the examined population and the retrospective nature of the study.

The predefined response options for why patients chose pessary therapy were based on clinical experience and observations. While this approach ensured practicality and consistency, it may have limited the ability to capture less common or nuanced patient perspectives. Including open-ended response options in future research could provide richer qualitative insights and a broader understanding of patient motivations.

No adjustments for potential confounding variables were made in this study. While the analysis focused on direct associations, the lack of multivariable adjustment may have influenced the results. Future research should incorporate multivariable analyses to account for potential confounders and provide more robust conclusions.

An additional limitation of this study is that women’s satisfaction with pessary use was not assessed using a validated questionnaire, furthermore the selected participants were women who continued to wear the pessary, excluding those who discontinued its use.”

## Conclusions

Our study provides a comprehensive analysis of demographic, socioeconomic, and functional health-related factors influencing pessary choice for the conservative treatment of pelvic organ prolapse (POP). Our findings emphasize underexplored aspects such as hand function, vision, mobility, and sexual activity in pessary selection. Notably, the preference for daily control pessaries (DCP) among younger, sexually active patients and for long-term wear pessaries (LTP) among older, less mobile patients underscores the importance of tailoring treatment to individual characteristics. By incorporating functional parameters like hand dexterity and mobility into decision-making, this study offers actionable insights to bridge critical knowledge gaps and improve patient-centered care in POP management. The results of our study confirm our hypothesis that a number of demographic, socio-economic and functional health-related factors plays a role in choosing between long-term wear and daily control pessaries for the conservative treatment of POP. Factors such as anatomical findings, the severity of the condition, general health status, and patient opinion and preferences influence the choice of pessary type.

Patients who do not want or are unable to manage a DCP due to impaired hand function, vision, or mobility may benefit from a LTP since they do not require daily removal and reinsertion.

## Data Availability

The data that support the findings of this study are available from the authors, but restrictions apply to the availability of these data to protect study participant privacy and so are not publicly available. Data are, however, available from the authors upon reasonable request. (Orsolya Oláh, email: olah.orsolya@med.semmelweis-univ.hu)

## Abbreviations

POP: Pelvic organ prolapse
DCP: Daily control pessary
LTP: Long-term wear pessary
DC: Daily control
LT: Long term wear
